# Mindfulness as a key mechanism: linking flow and emotional intelligence to academic engagement in Chinese university students

**DOI:** 10.3389/fpsyg.2025.1635604

**Published:** 2025-10-28

**Authors:** You Wu, Rui Ma

**Affiliations:** ^1^Dazhou Vocational and Technical College, Dazhou, Sichuan, China; ^2^School of Health and Rehabilitation, Chengdu University of Traditional Chinese Medicine, Chengdu, Sichuan, China

**Keywords:** academic flow, trait emotional intelligence, mindfulness, academic engagement, psychological mechanisms, student wellbeing, state-trait dynamics

## Abstract

**Introduction:**

This study investigates the interrelationships among academic flow, trait emotional intelligence (EI), and mindfulness as predictors of academic engagement among Chinese university students. We explore these dynamics at both trait and momentary state levels, a gap in the existing literature.

**Methods:**

A multi-phase mixed-methods design was employed. The initial phase involved quantitative surveys with 394 university students. A subset of 30 students participated in qualitative focus group discussions, followed by an Experience Sampling Method (ESM) phase with 80 students over 7 days. Data were analyzed using structural equation modeling (SEM) for the survey data, multilevel modeling (MLM) for the ESM data, and thematic analysis for the qualitative data.

**Results:**

SEM results showed that trait academic flow and trait EI had significant positive direct effects on academic engagement. Importantly, trait mindfulness emerged as a significant mediator of both these relationships, with the full model explaining 62% of the variance in engagement. MLM of the ESM data revealed that momentary mindfulness positively predicted subsequent increases in momentary academic flow and engagement. Additionally, higher trait EI was found to buffer the negative impact of momentary stress on real-time engagement. Thematic analysis of the focus groups provided rich contextual insights that supported these quantitative findings.

**Conclusion:**

The combined findings demonstrate that mindfulness is a critical psychological mechanism linking flow and emotional intelligence to academic engagement, functioning both as a stable disposition and a dynamic state. This multi-level evidence suggests that interventions aimed at enhancing mindfulness could be highly effective in fostering greater student engagement and success in higher education.

## 1 Introduction

Student engagement—the investment of emotion, cognition, and behavior in learning—strongly predicts academic success and wellbeing in higher education (Finn and Zimmer, 2012; Groccia, 2018; Schaufeli et al., 2002; Wong and Liem, 2022). Yet, students often face academic pressures and emotional setbacks that hinder their ability to stay engaged (Finn and Zimmer, 2012). This has spurred interest in psychological resources that may help students sustain their engagement, and how these operate both as stable traits and as dynamic states in daily academic life. Among these, academic flow, emotional intelligence (EI), and mindfulness appear promising (Brown and Ryan, 2003; Petrides et al., 2004; Shernoff et al., 2003).

One such resource, academic flow, involves deep immersion and focused energy during a task (Csikszentmihalyi, 1988), enhancing motivation and learning (Shernoff et al., 2003). It often occurs when task challenges match student skills, preventing boredom or anxiety (Fong et al., 2015). While flow is linked to academic success (Buil et al., 2019; Rossin et al., 2009), its specific contribution to engagement, particularly the momentary processes driving this connection, warrants further exploration. Similarly, trait emotional intelligence (EI)—the ability to perceive, understand, and manage emotions (Petrides et al., 2004)—helps students handle academic stress, improve focus, and persist through difficulties (Mavroveli and Sánchez-Ruiz, 2011; Qualter et al., 2012). Like flow, however, its influence is often studied independently, and its combined effect with other resources on engagement, as well as how trait EI translates into effective momentary emotional regulation, remains less understood.

Mindfulness—awareness of the present moment without judgment (Brown and Ryan, 2003; Kabat-Zinn, 2003)—is another resource gaining attention. It can improve focus, emotional regulation, and stress reduction, thereby supporting engagement (Brown and Ryan, 2003; Kabat-Zinn, 2003; Khoury et al., 2013; Medvedev et al., 2018). Researchers suggest mindfulness might enable flow by sharpening focus (Ergas and Hadar, 2019; Roeser et al., 2022) and could link EI to engagement through better emotional management (Grossman et al., 2004; McDonough and Lemon, 2018)—mechanisms that can be precisely examined through momentary assessments of these states and their interplay.

Although research highlights the individual benefits of flow, EI, and mindfulness, their combined influence on academic engagement, particularly at different timescales, remains underexplored. Previous studies have often treated these variables as stable traits (Brown and Ryan, 2003; Petrides et al., 2004; Shernoff et al., 2003), limiting insight into their synergistic effects and the real-time dynamics through which these influences unfold. A particular gap exists concerning the potential mediating role of mindfulness in the flow-engagement and EI-engagement relationships. Furthermore, while traditional qualitative methods provide insights into general lived experiences, there is a need to understand how these psychological resources fluctuate and interact within individuals’ daily academic routines and specific contexts.

To address this gap, the present study aims to investigate the interplay between academic flow, trait emotional intelligence (EI), and mindfulness in predicting academic engagement among Chinese university students. Our central research question is: To what extent does mindfulness function as a psychological mechanism that mediates the relationship between both academic flow and emotional intelligence on academic engagement? We explore this question using a multi-phase, mixed-methods design to capture both stable traits and dynamic, momentary states. Based on the literature, we primarily hypothesized that: (1) academic flow and trait EI would positively predict academic engagement, and (2) that mindfulness would mediate these relationships. By examining these dynamics at multiple levels of analysis, this study seeks to provide a comprehensive and ecologically valid understanding of the key drivers of student success in higher education.

## 2 Literature review and hypotheses development

### 2.1 Understanding academic engagement

Academic engagement, crucial in higher education, is defined as students’ active and purposeful involvement in learning, encompassing cognitive and emotional dimensions (Bowden et al., 2021; Finn and Zimmer, 2012; Kahu, 2013; Skinner and Pitzer, 2012). Beyond mere attendance, it reflects a deep commitment influencing learning approaches, responses to challenges, and goal achievement (Groccia, 2018; Parsons and Taylor, 2011; Payne, 2017; Wang and Eccles, 2013; Wong and Liem, 2022). Consistently, engagement correlates with positive outcomes: enhanced performance, better retention, and improved wellbeing (Casuso-Holgado et al., 2013; Finn and Zimmer, 2012; Guo et al., 2022; Hodge et al., 2018; Vizoso et al., 2018; Wong et al., 2024).

The Utrecht Work Engagement Model offers a key framework, describing engagement as a positive state with vigor, dedication, and absorption (Schaufeli et al., 2002). Vigor is high energy and resilience; dedication is significance and enthusiasm; absorption is deep concentration (Dimitriadou et al., 2020; Schaufeli et al., 2002). This model informs higher education research on cognitive and emotional investment in learning (van Rooij et al., 2017). Other perspectives also highlight engagement’s multidimensionality: behavioral, emotional, and cognitive aspects (Finn and Zimmer, 2012; Heilporn et al., 2024; Korhonen et al., 2019). For instance, Finn and Zimmer (2012) specify behavioral engagement as participation, emotional engagement as reactions, and cognitive engagement as mental effort (Alrashidi et al., 2016). These dimensions are synergistic, showing engagement is a holistic learning commitment integrating action, emotion, and thought (Derakhshan and Fathi, 2024; Finn and Zimmer, 2012).

In higher education, academic engagement’s importance is amplified by its strong link to student success (Han, 2024; Pike et al., 2012). Engaged students achieve more and persist longer (Casuso-Holgado et al., 2013; Guo et al., 2022; Hodge et al., 2018). Engagement improves performance by fostering deeper processing and sustained focus (Vizoso et al., 2018; Wong et al., 2024). It also enhances belonging, crucial for retention (Rowe et al., 2023; van Rooij et al., 2017), and strengthens resilience to academic stress (Denovan et al., 2020; Moyano et al., 2023). Engaged students exhibit better motivation and emotional regulation, navigating challenges positively (Sanchez-Ruiz et al., 2024; Thomas and Allen, 2021). Emotional intelligence, self-efficacy, and mindfulness are key resources enhancing engagement by improving emotion management and focus (Acosta-Gonzaga, 2023; Martínez et al., 2019).

Academic engagement is dynamic, influenced by student traits and environment (Sanchez-Ruiz et al., 2024). Interventions boosting emotional intelligence, self-efficacy, and mindfulness can help sustain engagement amidst pressures (Martínez et al., 2019; Moyano et al., 2023). Mindfulness and emotional regulation practices can enhance sustained engagement, supporting long-term academic success and wellbeing (Moyano et al., 2023).

In conclusion, academic engagement is vital for university student success, impacting performance, retention, and wellbeing. Models like the Utrecht model and multidimensional views offer frameworks to understand its cognitive, emotional, and behavioral aspects. Research emphasizes fostering engagement through interventions improving emotional intelligence, self-efficacy, and resilience. Promoting academic engagement should be a priority for educators and researchers aiming to optimize higher education outcomes.

### 2.2 Academic flow in education

Academic flow, often described as a state of profound absorption and engagement in an activity, is characterized by deep concentration, a diminished sense of time, and a fading awareness of external distractions (Csíkszentmihályi, 1996; Shernoff, 2012). Drawing from Csikszentmihalyi’s (1988) seminal flow theory, this optimal psychological state is fundamentally about experiencing heightened focus, intrinsic enjoyment, and a sense of mastery over the task at hand. Within educational contexts, academic flow specifically refers to students’ deep engagement with their academic work, achieved when there is a perceived equilibrium between the academic challenge and their perceived skill level (Abuhamdeh, 2020; Shernoff et al., 2003). This state is typified by an intense concentration, a merging of action and awareness, and a rewarding sense of accomplishment upon task completion (Asakawa, 2010; Shernoff and Csikszentmihalyi, 2009). Experiencing academic flow is not merely about task completion, but about the quality of experience during learning, making academic pursuits inherently rewarding.

Several key conditions are recognized as essential for students to enter and sustain academic flow. Foremost among these is the balance between challenge and skill. Csikszentmihalyi’s (1988) foundational work emphasizes that flow is most likely to occur when individuals perceive a task as optimally challenging, neither too easy (leading to boredom) nor excessively difficult (inducing anxiety) (Csikszentmihalyi, 2014; Fong et al., 2015; Harris et al., 2023). When students encounter tasks that are appropriately matched to their abilities, they are more inclined to become deeply involved and experience flow (Mills and Fullagar, 2008; Whalen, 1998). Complementary to this balance, clear goals and immediate feedback are also critical enablers of flow. These elements provide students with a sense of direction, purpose, and the necessary information to adjust their efforts effectively, maintaining alignment with task demands in real-time (Abuhamdeh, 2020; Buil et al., 2019; Pearce, 2005). Such conditions collectively foster an environment where students can fully concentrate, minimize distractions, and sustain their engagement throughout the learning process, thereby maximizing the potential for flow experiences (Wu et al., 2021).

The positive impact of academic flow on various learning outcomes is well-documented in the literature. Research consistently demonstrates that experiencing flow is associated with enhanced academic performance, increased intrinsic motivation, and greater overall engagement in learning (Beard, 2015; Csikszentmihalyi, 2014; Lee, 2005; Peifer et al., 2022). For instance, studies have shown that students who frequently experience flow report higher levels of intrinsic motivation and achieve better academic results (Rossin et al., 2009; Shernoff et al., 2003). Furthermore, the use of interactive learning tools that promote flow experiences has been linked to enhanced student engagement and improved academic performance in university settings (Buil et al., 2019). These findings underscore the significant role of flow in not only making learning more enjoyable but also in directly contributing to academic success.

Flow is especially relevant for university students due to demanding academics. Flow theory emphasizes balanced challenge and skill to maintain motivation and prevent boredom or anxiety (Csikszentmihalyi, 2014; Fong et al., 2015). University students must balance task difficulty to sustain flow. Research on architecture students shows flow is strongest in challenging yet skill-appropriate projects (Mills and Fullagar, 2008; Shernoff and Csikszentmihalyi, 2009). Beyond performance and motivation, flow significantly boosts academic satisfaction. Students in flow report greater learning satisfaction (Joo et al., 2014; Park and Lee, 2018). This satisfaction arises from flow’s intrinsic rewards, transforming study from obligation to enjoyment (Asakawa, 2010; Guan, 2013; Whalen, 1998). Flow’s deep engagement and accomplishment enhance students’ academic fulfillment and wellbeing (Wang et al., 2020).

### 2.3 Emotional intelligence in academia

Trait Emotional Intelligence (EI), defined as a constellation of emotional self-perceptions, encompasses the ability to recognize, understand, and manage one’s own emotions, alongside effective social navigation (Baudry et al., 2018; Chamorro-Premuzic et al., 2007; Petrides et al., 2004). This construct is often understood through facets like mood attention (monitoring emotions), emotional clarity (understanding emotions), and mood repair (regulating emotions, especially negative ones) (Bar-On, 2000; Chow et al., 2011; Salovey et al., 1995). These facets collectively equip individuals with emotional agility, crucial for responding effectively to diverse personal and academic challenges.

In higher education, a primary benefit of trait EI lies in its facilitation of emotional regulation and enhanced academic engagement, both vital for academic achievement (Laborde et al., 2014; Maguire et al., 2017; Perera and DiGiacomo, 2013; Thomas and Allen, 2021). Higher trait EI enables students to better manage academic stress and negative emotions like frustration and anxiety, thus sustaining focus and persistence in their studies (Ferrando et al., 2011; Mavroveli and Sánchez-Ruiz, 2011). This effective emotional regulation cultivates a positive approach to academic challenges, fostering motivation and sustained engagement (Qualter et al., 2012). Research supports that emotionally intelligent students utilize effective self-regulation, boosting both academic performance and engagement (MacCann et al., 2020; Okwuduba et al., 2021; Perera and DiGiacomo, 2013; Zhoc et al., 2018; Zhoc et al., 2021). By adeptly navigating emotional fluctuations in academic life, these students maintain motivation and perseverance across demanding tasks, leading to improved academic outcomes.

Furthermore, emotional intelligence significantly fosters resilience among university students (Tortosa Martínez et al., 2023). The academic environment is often laden with stressors such as heavy workloads, deadlines, and exams, which can impede performance without effective coping mechanisms (Zhoc et al., 2020). EI empowers students to adopt a solution-focused approach to these stressors, enabling them to reframe stressful situations and maintain goal orientation (Goh and Kim, 2021; Perera and DiGiacomo, 2013). By effectively regulating emotional responses, students with high trait EI are better prepared to lessen emotional reactivity and manage academic demands (Petrides et al., 2018). This resilience is particularly critical in higher education, where students juggle multiple pressures. Supporting this, research indicates that emotionally intelligent students exhibit greater cognitive and affective engagement, enhancing their capacity to overcome academic hurdles and recover from setbacks (Maguire et al., 2017).

Beyond self-regulation and resilience, trait EI is instrumental in fostering positive interpersonal relationships within academia (Perera and DiGiacomo, 2015). Emotionally intelligent students tend to build stronger connections with peers and instructors, creating a more supportive learning environment (Estrada et al., 2021). These positive relationships are vital for academic success, providing crucial emotional and social support that buffers against academic stress (Estrada et al., 2021). For instance, EI enhances students’ ability to communicate and collaborate effectively with peers, improving engagement and study habits (Iqbal et al., 2022). In higher education, where collaborative learning is key, cultivating and maintaining positive relationships significantly contributes to academic success.

Consistently, research highlights trait EI as a robust predictor of academic performance in higher education. Emotionally intelligent students not only manage emotions better but also demonstrate greater resilience, self-regulation, and academic engagement (Perera and DiGiacomo, 2013). This comprehensive emotional skillset facilitates better academic outcomes, stronger relationships, and a more positive academic outlook (Ferrando et al., 2011). Meta-analytic evidence confirms that emotionally intelligent students achieve superior academic performance across diverse educational settings (MacCann et al., 2020). The ability to regulate emotions and maintain focus during academic stress is a key asset for excelling in both individual and group learning contexts. These findings underscore the significant value of fostering emotional intelligence through educational interventions to enhance student success (Maguire et al., 2017; Sanchez-Ruiz et al., 2013).

### 2.4 The academic utility of mindfulness

Mindfulness, the practice of present-moment awareness with non-judgmental acceptance of thoughts, emotions, and sensations, is increasingly recognized for its psychological and educational benefits (Brown and Ryan, 2003; Kabat-Zinn, 2003; Sauer et al., 2013). It involves focusing on current experiences without judgment or emotional reactivity (Chems-Maarif et al., 2025; Creswell, 2017), promoting emotional regulation, stress reduction, and better cognitive function (Baer, 2003; Shapiro et al., 2006). Consequently, mindfulness is gaining traction in education for its potential to improve student wellbeing and academic success (Schonert-Reichl and Roeser, 2016; Tomlinson et al., 2018; Zhang and Fathi, 2024).

In academics, mindfulness significantly boosts academic engagement by enhancing concentration (Maynard et al., 2017). Mindfulness practices lessen mind-wandering and improve focus, enabling sustained attention on academic tasks (Brown and Ryan, 2003; Roeser, 2014). This heightened attention is crucial in today’s distracting digital environments (Ergas and Hadar, 2019). Mindfulness aids students in managing distractions through improved cognitive control, enhancing engagement in lectures, discussions, and study (Eberth and Sedlmeier, 2012; Fathi et al., 2025; Khoury et al., 2013). This deeper engagement fosters stronger intrinsic motivation (Aherne et al., 2016; Bush, 2013).

Furthermore, mindfulness is known for its stress-reducing effects, highly relevant in the high-pressure academic context (Fathi et al., 2023; Grossman et al., 2004; Morone et al., 2012). Mindfulness-Based Interventions (MBIs) effectively reduce anxiety and depression, providing students with tools to manage academic stress (Kabat-Zinn, 2003; Kerrigan et al., 2017). For example, mindfulness training improved university students’ mental wellbeing by reducing stress and enhancing emotional balance (Kerrigan et al., 2017). Stress reduction improves both academic engagement and overall mental health, supporting academic excellence.

Beyond engagement and stress, mindfulness positively impacts academic performance (Miralles-Armenteros et al., 2021; Vorontsova-Wenger et al., 2021). Mindfulness enhances cognitive functions like attention, working memory, and cognitive flexibility—essential for academic success (Khoury et al., 2013; Lertladaluck et al., 2021; Whitfield et al., 2022). Dispositional mindfulness correlates with lower anxiety and depression, linking to better academic outcomes (Medvedev et al., 2018). Mindfulness aids in regulating negative emotions during academic pressure, improving performance. Mindfulness training also enhances executive functions critical for complex academic tasks (Cásedas et al., 2020; Chiesa et al., 2011). Improved working memory and attention from mindfulness programs translate to better academic performance (Bush, 2013), valuable in demanding higher education (Felver et al., 2016; Zhang et al., 2024).

Mindfulness may also facilitate flow and emotional regulation during academic tasks (Teper et al., 2013; Xie, 2022; Yeh et al., 2019). It promotes flow by fostering present focus, minimizing distractions, and sustaining attention (Ergas and Hadar, 2019). Mindfulness practice increases flow experiences, enhancing academic performance (Vidal-Meliá et al., 2022), by encouraging task concentration and reducing rumination or worry (Roeser et al., 2022). Additionally, mindfulness significantly improves emotional regulation, crucial for academic success (Hill and Updegraff, 2012; Maynard et al., 2017; Roemer et al., 2015). It helps university students manage emotional challenges by fostering acceptance and composure (Bóo et al., 2020; McDonough and Lemon, 2018), building emotional resilience for a positive academic attitude even amidst setbacks (Baumgartner and Schneider, 2021; Grossman et al., 2004). Enhanced emotional regulation through mindfulness improves both academic performance and overall wellbeing.

### 2.5 Research aims and hypotheses

This multi-phase mixed-methods study aims to explore the interrelationships between academic flow, trait emotional intelligence (EI), mindfulness, and academic engagement among Chinese university students, examining these constructs at both stable trait levels and as dynamic, momentary states. While each factor is individually studied, their combined influence on student engagement, particularly the processes through which these influences unfold in daily academic life, remains unclear. This research addresses this gap by examining how these psychological factors interact and jointly affect academic engagement. Specifically, for the initial trait-level analysis, we aim to determine the direct effects of academic flow and EI on engagement, and mindfulness’s mediating role in these relationships.

Literature indicates that academic engagement—emotional, cognitive, and behavioral investment in learning—is key to student success (Groccia, 2018; Schaufeli et al., 2002). Fostering engagement requires understanding factors that enhance and sustain it. Academic flow, characterized by deep immersion, is linked to motivation, satisfaction, and performance (Csikszentmihalyi, 1988; Shernoff et al., 2003). Similarly, trait EI, involving emotion regulation, is tied to better academic outcomes through stress management and resilience (Ferrando et al., 2011; Petrides et al., 2004). However, the interplay of these constructs in influencing academic engagement, and how these trait-level associations manifest at a momentary level, is not fully understood.

Mindfulness, defined by present-moment awareness and non-judgmental acceptance (Brown and Ryan, 2003; Kabat-Zinn, 2003), facilitates emotional regulation and cognitive control in academic settings. Research suggests mindfulness enhances focus, reduces stress, and deepens engagement by improving emotion and attention management (Khoury et al., 2013; Medvedev et al., 2018). We propose mindfulness mediates the effects of both academic flow and EI on engagement, helping students manage emotions and maintain focus in challenging situations (Bush, 2013; Vidal-Meliá et al., 2022).

Complementing the initial quantitative findings, the initial qualitative aspect of this study (focus groups) will explore students’ general lived experiences with academic flow, EI, and mindfulness. While trait-level quantitative data provides general trends, this initial qualitative data will illuminate how these processes manifest in academic life, offering nuanced insights into students’ emotional and cognitive challenges in sustaining engagement from their reflective perspective.

Furthermore, the Experience Sampling Method (ESM) phase of this study specifically aims to: (1) Capture the dynamic, within-person relationships between momentary states of academic flow, emotional experience, mindfulness, and academic engagement as they occur in students’ daily lives (e.g., examining if momentary mindfulness predicts subsequent momentary flow). (2) Investigate how individual differences in trait-level flow, EI, and mindfulness (from the initial survey) relate to average momentary experiences and moderate these within-person dynamic processes (e.g., exploring if trait EI buffers the impact of momentary stress on engagement). (3) Explore the influence of specific academic contexts (e.g., activity type, location, social setting) on students’ momentary psychological states.

Based on the literature supporting trait-level relationships, this study hypothesizes for the initial SEM analysis: (H1) Academic flow will positively impact academic engagement, linked to focus and motivation (Csikszentmihalyi, 1988; Shernoff et al., 2003). (H2) Trait EI will positively impact academic engagement through emotional regulation and coping (Petrides et al., 2004; Qualter et al., 2012). (H3) Mindfulness will mediate the flow-engagement relationship by enhancing focus (Brown and Ryan, 2003; Roeser et al., 2022). (H4) Mindfulness will mediate the EI-engagement relationship by facilitating emotional regulation and reducing anxiety (McDonough and Lemon, 2018; Medvedev et al., 2018).

Drawing from theory and the established links at the trait level, we also formulated the following hypotheses for the ESM phase: (H5) Momentary mindfulness will positively predict concurrent and subsequent (lagged) momentary academic flow. (H6) Momentary mindfulness will positively predict concurrent and subsequent (lagged) momentary academic engagement. (H7) Momentary stress will negatively predict concurrent momentary academic engagement and flow. (H8) Momentary emotional regulation effectiveness will buffer the negative relationship between momentary stress and momentary academic engagement. (H9) Trait mindfulness, trait EI, and trait academic flow will each positively predict average levels of their corresponding state measures (i.e., average momentary mindfulness, emotional regulation effectiveness, and academic flow, respectively) during the ESM period. (H10) Trait EI will moderate the within-person negative relationship between momentary stress and momentary academic engagement, such that this relationship is weaker for students with higher trait EI. (H11) Academic context (e.g., studying alone versus in class, being in the library versus at home) will significantly influence momentary levels of academic flow and engagement.

Using this comprehensive, multi-phase mixed-methods approach, integrating survey, focus group, and ESM data, this study aims to provide a more holistic and ecologically valid understanding of how academic flow, EI, and mindfulness collectively influence engagement, contributing to both theory and practice in higher education.

## 3 Methodology

### 3.1 Research design

We employed a multi-phase mixed-methods design. The initial phase utilized a convergent parallel approach (Creswell and Plano Clark, 2018). This design involves collecting and analyzing quantitative (survey) and qualitative (focus group) data separately during the same timeframe. The results are then merged during the interpretation phase to create a more comprehensive and nuanced understanding of a research problem. This method was chosen to leverage both the generalizable trends from the survey data and the in-depth contextual insights from student discussions. Following this, an Experience Sampling Method (ESM) phase was implemented with a subset of participants to capture dynamic, in-situ experiences related to the core constructs.

### 3.2 Study participants

A total of 394 Chinese university students participated in this study. Students were meticulously recruited from four geographically diverse universities in China to ensure a representative sample across different regional and institutional contexts. The universities included a comprehensive research-intensive university in Eastern China, a technological university in Central China, a normal university with a strong humanities focus in Western China, and a university specializing in international business in Southern China. This selection strategy aimed to capture a broad range of academic disciplines and student experiences within the diverse Chinese higher education system. Participants were aged between 18 and 24 years (*M* = 20.3, SD = 1.4), with 176 males (44.7%) and 218 females (55.3%). The sample included students from different academic years to account for variations in academic experience and pressures, with 53.3% being sophomores (n = 210), 33.5% juniors (*n* = 132), and 13.2% either freshmen (*n* = 30) or seniors (*n* = 22).

Inclusion criteria required participants to be actively enrolled in a full-time undergraduate program at one of the participating universities and to provide informed consent to participate in both the quantitative survey and the subsequent qualitative interview phases of the study. To ensure a diverse representation of academic backgrounds, students’ self-reported academic performance (measured by GPA, with a range observed from 2.5 to 4.0 on a 4.0 scale) and academic major were recorded. The sample included students from a wide array of disciplines, including engineering (25.3%), humanities and social sciences (29.7%), natural sciences (20.1%), business and economics (14.9%), and education (9.9%).

From the initial 394 participants, a subset of *N* = 80 students was subsequently invited to participate in the ESM phase. This subset was selected using stratified sampling based on their initial survey responses for academic major and year level to ensure diverse representation. All ESM participants provided additional informed consent specifically for this intensive data collection phase.

We used a convenience sampling method for initial recruitment, stratified by gender, academic year, and major. Recruitment involved class announcements, online forums, and campus posters. Power analysis confirmed the initial sample size (*N* = 394) was adequate for SEM. Ethical approval for all study phases, including the ESM component, was obtained from the Ethical Review Board of Chengdu University of Traditional Chinese Medicine. Participants received full details about the study’s purpose, procedures, and their right to withdraw. All data were anonymized and stored securely.

### 3.3 Instrumentation

#### 3.3.1 Academic flow measure

Academic flow was measured using the Chinese version of the Academic Flow Questionnaire (Yuwanto, 2018), administered as part of the initial survey. This 14-item scale assesses flow in academic contexts through three dimensions: concentration, academic satisfaction, and intrinsic motivation. Participants rated items such as “I feel immersed in my academic tasks” on a 6-point Likert scale (1 = strongly disagree to 6 = strongly agree), with higher scores indicating greater academic flow. This questionnaire captures trait-level academic flow, reflecting a student’s typical tendency to experience flow in academic settings rather than momentary states. In this study, the scale showed good reliability (Cronbach’s *α* = 0.87) and confirmatory factor analysis supported its validity (χ^2^/df = 2.15, CFI = 0.95, RMSEA = 0.052).

#### 3.3.2 Trait EI measure

Trait emotional intelligence (EI) was measured using the Chinese version of the Trait Meta-Mood Scale (TMMS-24), originally developed by Salovey et al. (1995). This 24-item scale evaluates three key facets of emotional intelligence: mood attention (awareness of one’s emotions), emotional clarity (the ability to understand and distinguish between emotions), and mood repair (the capacity to regulate one’s emotions). Participants rated their agreement with each statement on a 5-point Likert scale (1 = strongly disagree, 5 = strongly agree). Sample items include “I think about my mood constantly” (mood attention), “I am usually very clear about my feelings” (emotional clarity), and “Although I am sometimes sad, I have a mostly optimistic outlook” (mood repair). In this study, the TMMS-24 demonstrated high internal consistency, with overall reliability reported at 0.90, and the individual subscales showing reliability scores of 0.88 (mood attention), 0.89 (emotional clarity), and 0.85 (mood repair). The construct validity of the TMMS-24 in this study was further supported by CFA results, indicating a good model fit: χ^2^/df = 2.55, CFI = 0.94, TLI = 0.93, RMSEA = 0.060 (90% CI [0.052, 0.068]), RMSR = 0.055.

#### 3.3.3 Mindfulness measure

Mindfulness was assessed using the Chinese version of the Mindful Attention Awareness Scale (MAAS), developed by Brown and Ryan (2003). This 15-item instrument captures the extent to which individuals are focused on and aware of the present moment in a non-judgmental manner. Responses were recorded on a 6-point Likert scale, with options ranging from “almost never” (6) to “almost always” (1), where higher scores indicate greater levels of mindfulness. Example items include “I find myself concentrating on the same unimportant thoughts over and over” and “I work automatically without being aware of what I’m doing,” both of which are reverse-scored. The MAAS has been widely used in academic settings and has consistently demonstrated high internal reliability, with a Cronbach’s alpha of 0.91 in this study. To ensure its applicability in the present study, CFA was conducted, revealing satisfactory construct validity for the MAAS: χ^2^/df = 2.80, CFI = 0.93, TLI = 0.92, RMSEA = 0.065 (90% CI [0.057, 0.073]), SRMR = 0.059.

#### 3.3.4 Academic engagement measure

Academic engagement was measured using the Chinese version of the Utrecht Work Engagement Scale for Students (UWES-S), adapted by Gan et al. (2007) from the original UWES-S developed by Schaufeli et al. (2006). This 17-item scale consists of three subscales: Vigor (students’ energy and enthusiasm for their academic work), Dedication (the sense of significance and pride in one’s academic tasks), and Absorption (full immersion and concentration in study activities). Participants responded on a 7-point Likert scale, ranging from “never” (0) to “every day” (6), with higher scores indicating greater academic engagement. Sample items include “I feel strong and vigorous when I study” (Vigor) and “I am enthusiastic about my studies” (Dedication). The scale has demonstrated excellent reliability in prior studies, with a Cronbach’s alpha of 0.93 for the full scale. The factor structure of the UWES-S was evaluated using CFA, which confirmed a well-fitting model and supported its construct validity in this research: χ^2^/df = 2.35, CFI = 0.95, TLI = 0.94, RMSEA = 0.055 (90% CI [0.048, 0.062]), RMSR = 0.048.

#### 3.3.5 Translation and adaptation of measures

All instruments used in the survey (Academic Flow Questionnaire, TMMS-24, and MAAS) were originally developed in English. To ensure their psychometric integrity and cultural appropriateness for our Chinese student sample, a rigorous translation and validation process was followed.

A standard back-translation procedure was implemented. First, two independent bilingual researchers with expertise in psychology and education translated the original English versions of each scale into Chinese. These initial translations were then reviewed by a third expert to resolve discrepancies and synthesize a single, culturally adapted Chinese version. Next, a different bilingual expert, blind to the original English scales, back-translated the Chinese version into English. The research team compared this back-translated version to the original English scales to identify and correct any subtle semantic or conceptual inconsistencies. This iterative process continued until a final Chinese version was agreed upon that maintained the conceptual equivalence of the original instruments.

Prior to the main study, a pilot test with a separate group of 35 Chinese university students was conducted to assess the clarity and comprehension of the translated items. Feedback from this pilot was used to make minor wording adjustments to ensure that the language was natural and easily understood by the target population, reflecting the specific academic and cultural context. These validation steps, including the CFA results presented in the respective sections, confirm that the Chinese versions of the scales are both reliable and valid for measuring these constructs among Chinese university students.

#### 3.3.6 Focus group discussions

To complement the quantitative data, we conducted five focus group discussions with a total of 30 students. The planning and development of these sessions followed a structured protocol to ensure consistency and depth.

First, participants were purposefully selected from the initial survey sample (*N* = 394) to represent a spectrum of experiences; we invited students who reported high, moderate, and low levels of academic engagement to ensure a diverse range of perspectives. Participants were then organized into five groups, each consisting of six students. Groups were composed of students from similar academic years (e.g., a group of sophomores, a group of juniors) to foster a comfortable and relatable environment for sharing experiences.

Each discussion was planned to last between 60 and 75 min and was conducted in a private, quiet meeting room on campus to ensure confidentiality. A trained moderator, who was not involved in the students’ academic assessment, facilitated each session using a semi-structured interview guide. This guide was developed based on the study’s core theoretical constructs (flow, EI, mindfulness, engagement) and was piloted with a separate group of students to refine the clarity and flow of the questions. Key topics and example prompts included exploring students’ experiences of immersion in academic tasks (“Can you describe a time when you felt fully immersed in a task during class?”) and the role of emotions in their studies (“How do you think your ability to manage your emotions affects your engagement with academic work?”).

With informed consent from all participants, each session was audio-recorded. The recordings were then transcribed verbatim to ensure data accuracy for the subsequent thematic analysis. This systematic approach allowed us to gather rich, contextual insights into the personal and emotional experiences of academic engagement, providing depth to the quantitative findings.

#### 3.3.7 ESM protocol and measures

For the ESM phase, we designed a concise momentary questionnaire to measure state-level psychological constructs and their context, using a 7-point Likert scale (1 = Not at all to 7 = Very much) unless otherwise noted. Items were adapted from validated scales or established ESM measures and refined for clarity, relevance, and quick completion through a pilot test with 10 students. State academic flow was assessed with two items (e.g., “Right now, I am completely immersed in what I am doing,” “Right now, I feel my skills are well-matched to this task”), showing good within-person consistency (*r* = 0.72) and person-mean reliability (*α* = 0.75). A single item measured state mindfulness (e.g., “Right now, I am aware of my thoughts and feelings without getting carried away by them”), chosen for its face validity and common use in ESM studies, with pilot testing confirming its clarity. State emotional experience and regulation were evaluated with five items: four asking “Right now, how [stressed/anxious/happy/motivated] do you feel?” and one asking “How well are you managing these feelings right now?” These standard ESM items demonstrated face validity, supported by pilot feedback. State academic engagement used two items (e.g., “How focused are you on your current academic task?” “How interested are you in what you are doing right now?”), with a within-person correlation of *r* = 0.68 and person-mean reliability of *α* = 0.71, indicating reasonable consistency. Contextual details were collected by asking participants their current activity (e.g., In class, Studying alone, Break/Relaxing), location (e.g., Library, Home, Outdoors), and social interaction status (Yes/No, specifying Peers, Faculty, etc., if applicable).

Furthermore, to assess the stability of momentary measures, we calculated the person-level reliability for multi-item state constructs (flow, engagement) using an approach analogous to Cronbach’s alpha, averaging across individuals’ momentary responses where appropriate. These person-mean reliabilities were deemed acceptable (e.g., state flow person-mean α = 0.75; state engagement person-mean α = 0.71). The single-item mindfulness and emotional regulation effectiveness measures relied on their established face validity and derivation from existing ESM literature, supported by pilot testing for clarity in the current study context.

### 3.4 Data collection process

Data collection occurred over an extended period during the spring 2024 semester. Initial recruitment involved faculty collaboration and campus/online announcements, with interested students registering via an online form.

The initial quantitative survey phase (for all *N* = 394 participants) took place during weeks 2–4. Participants completed surveys (~45 min) via Qualtrics or Wenjuan.com in supervised computer labs. A pilot study (*N* = 35) informed minor instrument wording adjustments. The initial qualitative focus groups (with the selected *N* = 30) were conducted during weeks 10–12, as previously described.

The ESM data collection phase with the invited subset of *N* = 80 students occurred during weeks 14–15. Participants were prompted six times per day for seven consecutive days via a dedicated ESM smartphone application (e.g., movisensXS). Prompts were delivered semi-randomly between 9:00 a.m. and 9:00 p.m., with a minimum inter-prompt interval of 90 min. Each momentary survey was designed to be completed in 1–2 min. Prior to the ESM phase, participants attended a mandatory training session to install the app, understand the response protocol, and address any queries. A separate pilot test of the ESM protocol (*N* = 10 students not in the main ESM sample) was conducted for 2–3 days to refine prompt timing and item clarity. During ESM data collection, compliance was monitored, and technical support was available.

### 3.5 Analytical approach

The analysis for this study was conducted in multiple stages corresponding to the design. The quantitative data collected through the online survey platform were first exported to SPSS 28 for preliminary descriptive analysis, which included calculating means, standard deviations, and reliability indices for each of the scales used. Pearson’s correlations were performed to explore the initial relationships between the variables. Before conducting the SEM, we also assessed potential issues with multicollinearity among the exogenous (predictor) variables. To further investigate the hypothesized mediation model, where mindfulness was posited as a mediator between academic flow and academic engagement, structural equation modeling (SEM) was conducted using AMOS 27. SEM was chosen because it allows for the simultaneous testing of multiple relationships and provides a robust method for assessing the direct and indirect effects of variables (Kline, 2011). The model fit was evaluated using commonly accepted fit indices, including the chi-square (χ^2^), comparative fit index (CFI), Tucker-Lewis index (TLI), root mean square error of approximation (RMSEA), and standardized root mean square residual (SRMR). Good model fit was indicated by a CFI and TLI above 0.90, an RMSEA below 0.06, and an SRMR below 0.08 (Hu and Bentler, 1999; Marsh et al., 2004).

In addition to SEM, a bootstrapping procedure with 5,000 resamples was used to test the significance of the indirect effects of academic flow on engagement via mindfulness. Bootstrapping provides a non-parametric way to assess mediation and is particularly useful in cases where the sample size is not exceedingly large (Preacher and Hayes, 2008). This approach enabled a more accurate estimate of the confidence intervals for the indirect effects.

For the qualitative data, the focus group recordings were transcribed verbatim and imported into NVivo 12 for thematic analysis (Braun and Clarke, 2006). Two independent coders conducted the initial coding to ensure inter-rater reliability, with a Cohen’s kappa of 0.82 indicating substantial agreement. Themes were identified through an inductive approach, meaning that the analysis was driven by the data rather than predefined theoretical constructs. Key themes that emerged included the role of intrinsic motivation in sustaining flow, the emotional challenges students faced in maintaining engagement, and the impact of mindfulness on their ability to concentrate in academic settings. These qualitative findings were used to complement the quantitative results, providing deeper insight into how students’ emotional and cognitive experiences interacted to influence their overall academic engagement. The combination of quantitative correlations and qualitative narratives allowed for a more nuanced understanding of the complex relationships between the studied variables, contributing to the validity and richness of the research findings.

For the ESM data, analysis involved several steps. First, data were pre-processed, including structuring into a ‘long’ format (multiple observations nested within individuals) and addressing missing momentary responses. Subsequently, Multilevel Modeling (MLM) using HLM 8 was employed to examine within-person processes (e.g., how momentary mindfulness predicts subsequent momentary flow) and between-person differences (e.g., whether individuals with higher trait EI show different patterns of momentary emotional regulation). We also planned to explore cross-level interactions, such as whether trait mindfulness (from the initial survey) moderated within-person relationships between momentary stress and engagement.

Finally, findings from the initial quantitative SEM, the initial qualitative focus groups, and the ESM phase (both quantitative MLM results and any qualitative follow-up based on ESM patterns, if conducted) were triangulated and integrated during the overall interpretation to provide a comprehensive and nuanced understanding of the relationships between academic flow, EI, mindfulness, and academic engagement.

## 4 Findings

### 4.1 Quantitative results

#### 4.1.1 Data pre-processing

We began by examining the dataset (*N* = 394) for missing data, outliers, and normality. Missing data, comprising less than 5% of responses, were handled using the Expectation–Maximization (EM) algorithm, assuming data were missing at random (MAR) (Dempster et al., 1977). Four univariate outliers (∣*z*∣ > 3.29) were identified and winsorized to reduce their influence while preserving the data points; sensitivity analyses indicated this did not substantively change the results. Mahalanobis distance checks revealed no multivariate outliers (*p* < 0.001). While tests showed acceptable univariate normality (skewness < |2|, kurtosis < |7|), Mardia’s coefficient suggested slight multivariate non-normality. Given the sample size and the general robustness of Maximum Likelihood (ML) estimation under such conditions, we deemed ML appropriate for the subsequent structural equation modeling (SEM) (Kline, 2011; West et al., 1995).

To ensure the stability of the SEM, we also checked for multicollinearity among the predictor variables (academic flow, trait EI, and mindfulness). An examination of the tolerance and Variance Inflation Factor (VIF) values for these constructs revealed that all VIF values were well below the common threshold of 5 (VIFs ranged from 1.6 to 2.1) and tolerance values were above 0.10. Additionally, the bivariate correlations among the predictors (as shown in Table 1) were all below the conservative threshold of *r* = 0.80. These results indicate that multicollinearity was not a significant issue and that the model estimates were stable and reliable.

**Table 1:** Inter-correlations, composite reliability (CR), and average variance extracted (AVE).

Variable	CR	AVE	1	2	3	4
1. Academic flow	0.88	0.54	**0.73**			
2. Trait EI	0.90	0.58	0.48**	**0.76**		
3. Mindfulness	0.91	0.56	0.57**	0.50**	**0.75**	
4. Academic engagement	0.93	0.62	0.59**	0.53**	0.62**	**0.79**

Values on the diagonal (in bold) are the square roots of the AVEs. CR, composite reliability; AVE, average variance extracted. **p < 0.001.

#### 4.1.2 Descriptive analysis and bivariate correlations

Table 2 displays the descriptive statistics and Cronbach’s alpha (*α*) values. On average, students reported moderate-to-high levels for academic flow (*M* = 4.72, SD = 0.76), trait EI (*M* = 3.87, SD = 0.64), mindfulness (*M* = 4.10, SD = 0.58), and academic engagement (*M* = 5.12, SD = 0.88). All scales exhibited strong internal consistency, with α values ranging from 0.88 to 0.93.

**Table 2:** Descriptive statistics and reliability for the variables (*N* = 394).

Variable	Mean	SD	Cronbach’s *α*
Academic flow	4.72	0.76	0.88
Trait emotional intelligence (EI)	3.87	0.64	0.90
Mindfulness	4.10	0.58	0.91
Academic engagement	5.12	0.88	0.93

Pearson correlation analyses (Table 1) indicated significant, positive relationships among all key variables (*p* < 0.001). Academic engagement demonstrated moderate-to-large correlations with academic flow (*r* = 0.59), trait EI (*r* = 0.53), and mindfulness (*r* = 0.62), based on Cohen’s (1988) guidelines. Additionally, academic flow and mindfulness were positively correlated (*r* = 0.57). These results offer preliminary support for the hypothesized relationships (Table 3).

**Table 3:** Direct and indirect effects from structural equation model.

Effect type	Path	Standardized beta (*β*)	SE	*p*-value
Direct	Academic Flow → Academic Engagement	0.38	0.05	<0.001
Direct	Trait EI → Academic Engagement	0.29	0.04	<0.001
Direct	Mindfulness → Academic Engagement	0.42	0.06	<0.001
Indirect	Academic Flow → Mindfulness → Academic Engagement	0.24	0.03	<0.001
Indirect	Trait EI → Mindfulness → Academic Engagement	0.21	0.03	<0.001
Total effect (estimated)	Academic Flow → Academic Engagement (Total)	0.62	0.07	<0.001
Total effect (estimated)	Trait EI → Academic Engagement (Total)	0.50	0.06	<0.001
Proportion mediated (estimated)	Academic Flow → Mindfulness → Academic Engagement	39%		
Proportion mediated (estimated)	Trait EI → Mindfulness → Academic Engagement	42%		

#### 4.1.3 Measurement model

We first evaluated the measurement model using Confirmatory Factor Analysis (CFA) in AMOS 27. This step was essential to confirm that our observed variables effectively represented their intended latent constructs—academic flow, trait EI, mindfulness, and academic engagement—thereby establishing construct validity. The results indicated that the hypothesized four-factor model achieved a good fit with the data: *χ*^2^(120) = 210.65, *p* < 0.001; CFI = 0.955; TLI = 0.946; RMSEA = 0.046 (90% CI [0.034, 0.058]); and SRMR = 0.035. These fit indices meet established guidelines, supporting the model’s structure and providing a solid foundation for testing the structural relationships.

To further establish the reliability and validity of our measures, we assessed composite reliability (CR) and average variance extracted (AVE) for each latent construct. Composite reliability, which is often considered a more robust measure of internal consistency than Cronbach’s alpha in SEM, was high for all constructs, ranging from 0.88 to 0.94 and exceeding the recommended threshold of 0.70. For convergent validity, the AVE for each construct ranged from 0.52 to 0.63, all surpassing the 0.50 threshold (Fornell and Larcker, 1981), which confirms that a substantial portion of the variance in the indicator items is explained by their respective latent constructs.

Discriminant validity was assessed by comparing the square root of the AVE for each construct with its correlations with all other constructs. As shown in Table 1, the square roots of the AVEs (presented on the diagonal in bold) were all greater than the inter-construct correlations, providing evidence that each latent variable was distinct and measured a unique concept. For instance, the square root of the AVE for academic engagement (0.79) was greater than its highest correlation with any other variable (e.g., *r* = 0.62 with mindfulness), demonstrating adequate discriminant validity.

Furthermore, all indicator factor loadings exceeded 0.70, demonstrating strong convergent validity (Hair et al., 2010). To address potential common method bias (CMB) inherent in self-report studies, we also conducted Harman’s single-factor test. The first factor extracted in an exploratory analysis accounted for 29.4% of the total variance. Since this value is considerably below the 50% threshold often cited as a concern (Podsakoff et al., 2003), it suggests that CMB is unlikely to be a pervasive issue influencing our findings.

#### 4.1.4 Structural equation modeling

This study tested a structural equation model to examine whether mindfulness mediates the relationships between academic flow, trait emotional intelligence (EI), and academic engagement. Hypothesis 1 (H1) predicted a positive direct effect of academic flow on engagement, Hypothesis 2 (H2) predicted a similar effect for trait EI, Hypothesis 3 (H3) posited mindfulness as a mediator between flow and engagement, and Hypothesis 4 (H4) proposed mindfulness as a mediator between EI and engagement. The model showed good fit: *χ*^2^(144) = 258.23, *p* < 0.001, CFI = 0.956, TLI = 0.947, RMSEA = 0.045 (90% CI [0.035, 0.056]), SRMR = 0.038, indicating a robust representation of variable relationships.

**Figure 1:**
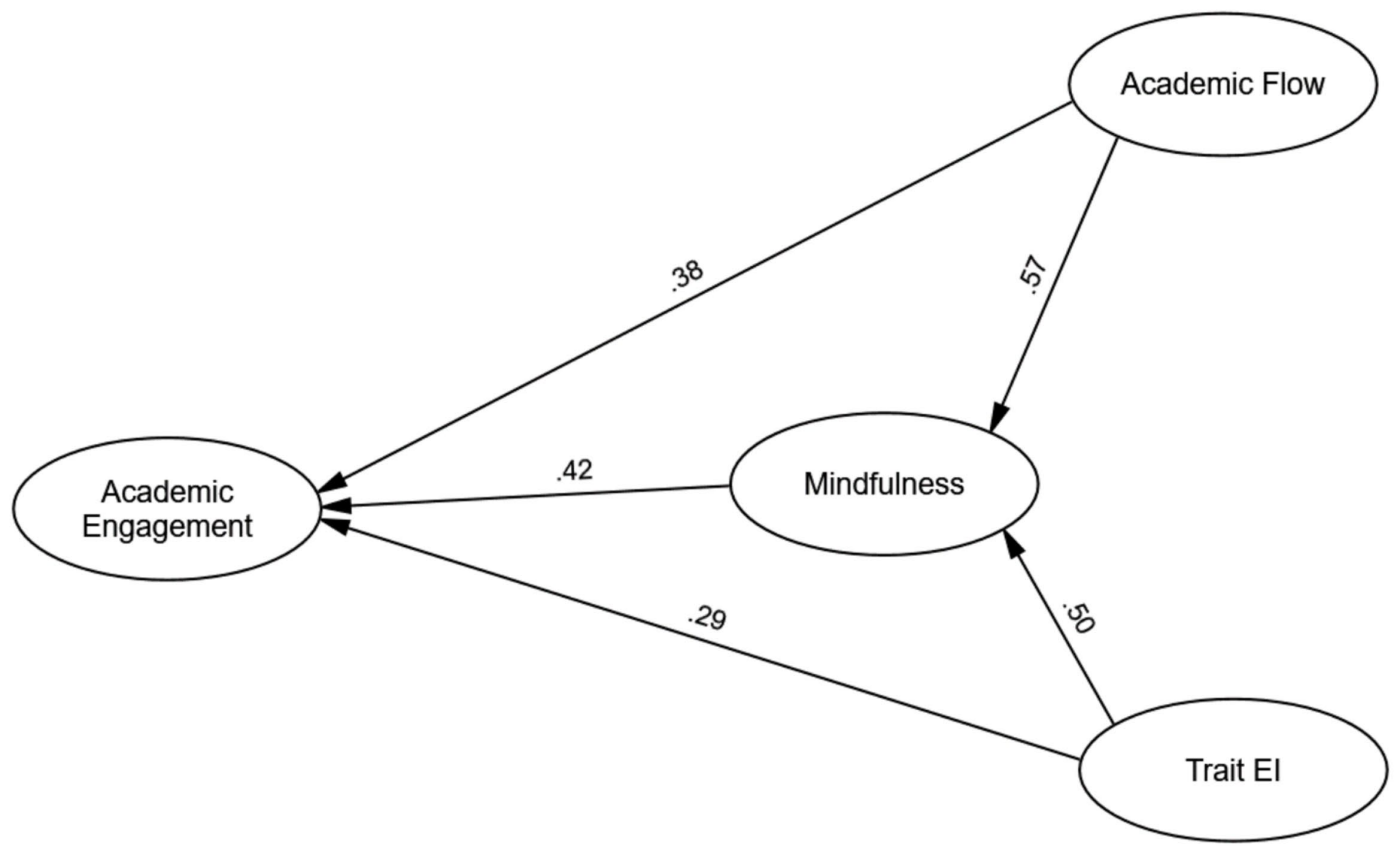
The mediation model of academic flow, trait emotional intelligence (EI), and mindfulness on academic engagement. Standardized path coefficients are shown. All paths are significant at *p* < 0.001.

Path analysis (Figure 1) confirmed significant direct effects. Academic flow positively influenced engagement (*β* = 0.38, *p* < 0.001), supporting H1, with a 1 SD increase in flow raising engagement by 0.38 SD. Trait EI also positively affected engagement (*β* = 0.29, *p* < 0.001), supporting H2. Mindfulness showed a strong direct effect on engagement (*β* = 0.42, *p* < 0.001), independent of flow and EI.

We tested the indirect effects using bootstrapping (5,000 samples), and the results supported our mediation hypotheses. Mindfulness mediated the flow-engagement relationship (*β* = 0.24, *p* < 0.001, 95% Bias-Corrected CI [0.18, 0.30]), confirming H3. Similarly, mindfulness mediated the EI-engagement relationship (*β* = 0.21, *p* < 0.001, 95% Bias-Corrected CI [0.15, 0.27]), supporting H4. As neither confidence interval included zero, both indirect effects were statistically significant. These findings highlight mindfulness as a key mediator, channeling significant portions of flow and EI’s effects on academic engagement.

The model explained 62% of the variance in academic engagement (*R*^2^ = 0.62), a large effect size per behavioral science standards. This indicates that academic flow, trait emotional intelligence (EI), and mindfulness strongly predict student engagement. The SEM results supported the hypothesized mediation model, confirming significant direct and indirect effects of flow and EI on engagement, with mindfulness as a key mediator. These findings highlight the critical role of direct and indirect pathways through flow, EI, and mindfulness in fostering engagement, underscoring their practical importance for enhancing student outcomes in higher education.

#### 4.1.5 Experience sampling method (ESM) results

The ESM phase involved *N* = 80 students responding to six daily prompts over 7 days, yielding 2,554 of 3,360 possible assessments (76.0% compliance). Participants averaged 31.93 (SD = 5.82) responses. Two participants with <30% compliance were excluded, resulting in *N* = 78 for multilevel modeling (MLM). An analysis of response patterns showed that missing responses were more common during evening hours (after 6:00 p.m.) and on weekends, possibly due to lower engagement or higher fatigue during these times. The overall compliance rate remained robust at 76.0%, and sensitivity analyses confirmed that results were consistent across missing data handling methods, including listwise deletion and multiple imputation.

Momentary variables (1–7 scales) showed moderate levels: state academic flow (*M* = 4.21, SD = 1.48), state mindfulness (*M* = 4.35, SD = 1.51), and state academic engagement (*M* = 4.62, SD = 1.65). Momentary motivation (*M* = 4.50, SD = 1.70) and happiness (*M* = 4.80, SD = 1.55) exceeded stress (*M* = 3.85, SD = 1.60) and anxiety (*M* = 3.50, SD = 1.58). Intra-class correlations for state flow (ICC1 = 0.42) and engagement (ICC1 = 0.38) supported MLM due to between-person variability.

MLM used HLM 8 (Level 1: momentary, person-mean centered; Level 2: individuals, grand-mean centered), controlling for time and day (see Table 4). At Level 1, momentary mindfulness predicted concurrent flow (*γ* = 0.37, SE = 0.03, *t*(2,400) = 12.33, *p* < 0.001) and engagement (*γ* = 0.43, SE = 0.04, *t*(2,400) = 10.75, *p* < 0.001). This suggests that a one-unit increase in momentary mindfulness (e.g., feeling more aware and present) is linked to a 0.37-unit rise in flow, reflecting a moderate increase in students’ focus and immersion during academic tasks. Prior mindfulness (*t* − 1, 90–180 min earlier) predicted subsequent flow (*γ* = 0.20, SE = 0.03, *t*(2,320) = 6.67, *p* < 0.001) and engagement (*γ* = 0.24, SE = 0.03, *t*(2,320) = 8.00, *p* < 0.001), indicating a lasting but slightly reduced effect on engagement over short intervals. Stress reduced concurrent engagement (*γ* = −0.30, SE = 0.04, *t*(2,400) = −7.50, *p* < 0.001) and flow (*γ* = −0.27, SE = 0.03, *t*(2,400) = −9.00, *p* < 0.001), but effective emotional regulation weakened this effect (*γ* = 0.12, SE = 0.04, *t*(2,398) = 3.00, *p* = 0.003). This interaction shows that students with stronger emotional regulation skills experience a smaller drop in engagement under stress, pointing to a protective effect.

**Table 4:** Multilevel modeling results for momentary academic flow, engagement, and related constructs (*N* = 78).

Effect type	Predictor	Outcome	*γ*	*SE*	*t*	df	*p*
Level 1: within-person effects							
Contemporaneous	Momentary Mindfulness	State Academic Flow	0.37	0.03	12.33	2,400	<0.001
Contemporaneous	Momentary Mindfulness	State Academic Engagement	0.43	0.04	10.75	2,400	<0.001
Lagged	Prior Momentary Mindfulness (*t* − 1)	State Academic Flow	0.20	0.03	6.67	2,320	<0.001
Lagged	Prior Momentary Mindfulness (*t* − 1)	State Academic Engagement	0.24	0.03	8.00	2,320	<0.001
Contemporaneous	Momentary Stress	State Academic Engagement	−0.30	0.04	−7.50	2,400	<0.001
Contemporaneous	Momentary Stress	State Academic Flow	−0.27	0.03	−9.00	2,400	<0.001
Interaction	Momentary Stress × Emotional Regulation	State Academic Engagement	0.12	0.04	3.00	2,398	0.003
Level 2: between-person effects							
Main Effect	Trait Mindfulness	Average State Mindfulness	0.58	0.06	9.67	76	<0.001
Main Effect	Trait EI	Average Emotional Regulation	0.51	0.05	10.20	76	<0.001
Main Effect	Trait EI	Average Momentary Stress	−0.33	0.07	−4.71	76	<0.001
Cross-Level Interaction	Trait EI × Momentary Stress	State Academic Engagement	0.16	0.05	3.20	2,398	0.001
Cross-Level Interaction	Trait-Level Academic Flow × Momentary Mindfulness	State Academic Flow	0.14	0.04	3.50	2,318	<0.001
Contextual effects (Level 1)							
Activity	Studying Alone vs. In Class	State Academic Flow	0.33	0.05	6.60	2,350	<0.001
Location	Library vs. Home	State Academic Engagement (Focus)	0.26	0.04	6.50	2,350	<0.001
Location	Outdoors vs. Indoors (During Break)	Positive Emotions	0.29	0.06	4.83	450	<0.001

Multilevel models were estimated using HLM 8, with Level 1 predictors person-mean centered and Level 2 predictors grand-mean centered. Models controlled for time of day and day of the week. *γ*, unstandardized regression coefficient; SE, standard error; *t*, *t*-statistic; df, degrees of freedom; *p*, *p*-value.

At Level 2, trait mindfulness predicted higher state mindfulness (*γ* = 0.58, SE = 0.06, *t*(76) = 9.67, *p* < 0.001). Trait EI enhanced emotional regulation (*γ* = 0.51, SE = 0.05, *t*(76) = 10.20, *p* < 0.001) and reduced stress (*γ* = −0.33, SE = 0.07, *t*(76) = −4.71, *p* < 0.001). Trait EI mitigated stress’s impact on engagement (*γ* = 0.16, SE = 0.05, *t*(2,398) = 3.20, *p* = 0.001), meaning that students with higher trait EI are less affected by stress, emphasizing emotional intelligence as a buffer in academic contexts. Students with higher trait-level academic flow, as measured by the Academic Flow Questionnaire in the initial survey, showed a stronger mindfulness-flow link (*γ* = 0.14, SE = 0.04, *t*(2,318) = 3.50, *p* < 0.001), suggesting that those with a greater tendency to experience flow benefit more from mindfulness in enhancing their immersion.

Contextual effects included higher flow when studying alone versus in class (*γ* = 0.33, SE = 0.05, *t*(2,350) = 6.60, *p* < 0.001), greater focus in libraries than at home (*γ* = 0.26, SE = 0.04, *t*(2,350) = 6.50, *p* < 0.001), and more positive emotions outdoors versus indoors (*γ* = 0.29, SE = 0.06, *t*(450) = 4.83, *p* < 0.001). These ESM findings reveal dynamic, ecologically valid patterns in mindfulness, flow, and engagement, highlighting within-person processes, trait moderation, and contextual influences. They complement SEM results by capturing real-time fluctuations in students’ academic experiences.

### 4.2 Qualitative results

To deepen our understanding of the quantitative findings, we conducted focus group discussions with 30 university students. Through thematic analysis (Braun and Clarke, 2006), we identified three core themes describing their lived experiences: (1) Experiencing Academic Flow, (2) Emotional Regulation for Academic Resilience and Engagement, and (3) Mindfulness in Enhancing Engagement and Flow. An additional theme covering challenges also emerged.

#### 4.2.1 Experiencing academic flow

Participants often described Academic Flow as a state of deep immersion in academic tasks, characterized by intense focus and a distorted sense of time. Many emphasized that achieving flow required a balance between task challenge and their own skills. As one engineering student explained: *“When I’m coding and debugging, especially when it’s a really tough problem but I know I have the skills to solve it, it’s like entering another world. Time just melts away.”* (Participant 17, Male, Junior, STEM Major). This quote underscores the importance of tasks being engagingly difficult but not overwhelming.

Intrinsic motivation was also a frequently mentioned catalyst. Students found it easier to enter a flow state when working on tasks they perceived as inherently interesting or meaningful. A design student shared: *“It’s projects where I have creative freedom. It’s not just about the grade; it’s about bringing my own ideas to life, and that’s when I truly get lost in the work.”* (Participant 25, Female, Sophomore, Arts/Design Major).

However, students also noted the fragility of flow. External distractions, like *“a noisy library or the constant notifications on my phone,”* could *“instantly break my concentration. It’s like a bubble bursting.”* (Participant 9, Male, Sophomore, Humanities Major). Internal pressures and racing thoughts could also act as barriers, with one student expressing doubt: *“I wish I could experience this ‘flow’… but honestly, my mind races too much.”* (Participant 3, Female, Senior, Social Sciences Major). These accounts highlight that both optimal conditions and individual factors shape flow experiences.

#### 4.2.2 Emotional regulation for academic resilience and engagement

Students described how they used Emotional Intelligence (EI) to navigate academic stress and maintain their engagement. They saw EI as a key resource for resilience and perseverance. Emotional awareness—recognizing feelings—was seen as the critical first step: *“Recognizing when I’m feeling overwhelmed or anxious is crucial. If I ignore those feelings, they just build up…”* (Participant 28, Female, Senior, Social Sciences Major).

This awareness paved the way for effective coping, often involving emotional clarity (understanding feelings). Students mentioned strategies like cognitive reappraisal and seeking social support: *“When I get a bad grade… I try to understand why I did not do well… It’s about turning that negative emotion into something productive.”* (Participant 11, Male, Junior, STEM Major). Another student added: *“Talking to my friends or even my professors… They offer different perspectives and encouragement… makes me feel less alone and more motivated…”* (Participant 6, Female, Sophomore, Humanities Major).

Yet, participants acknowledged that EI has its limits, particularly when faced with intense external pressures. One noted: *“EI helps, sure, but it’s not a shield against everything… Sometimes, the system itself is just stressful.”* (Participant 22, Male, Senior, Technological University, STEM Major). This suggests that personal emotional skills interact significantly with broader environmental factors.

#### 4.2.3 Mindfulness in enhancing engagement and flow

Participants reported that mindfulness practices helped them engage more deeply and experience flow more often. They viewed mindfulness as a tool for improving attention and managing distractions: *“When my mind starts to wander during lectures… I use my breath as an anchor… It’s like hitting a mental reset button.”* (Participant 5, Female, Sophomore, Humanities Major).

Mindfulness was also linked to stress reduction and a more accepting mindset, which indirectly supported engagement. A senior student reflected: *“Exam periods used to be incredibly stressful… Now, I meditate… I feel calmer, less reactive, and more able to focus… And surprisingly, in that calmer state, I find myself getting more absorbed in the material.”* (Participant 30, Male, Senior, STEM Major).

Many perceived a direct connection between mindfulness and flow, suggesting it acted as a form of *“mental training for flow… It’s like clearing away the mental noise…”* (Participant 14, Female, Junior, Social Sciences Major). However, mindfulness was not a panacea; some found it challenging: *“I tried those mindfulness apps… I just ended up feeling more stressed because I could not quiet my mind. It felt like another thing I was failing at…”* (Participant 1, Female, Freshman, Arts/Design Major). This highlights that mindfulness is a skill requiring practice and individual fit.

#### 4.2.4 Additional insights: challenges to sustained engagement

Beyond these psychological resources, students voiced significant challenges. A fundamental barrier was a lack of interest in the subject matter: *“If I genuinely dislike a subject, no amount of mindfulness or emotional intelligence is going to make me engaged.”* (Participant 19, Male, Junior, Humanities Major). External pressures, like financial or family responsibilities, also played a major role: *“When you are worried about making rent, academic engagement feels like a luxury.”* (Participant 27, Female, Senior, Technological University, Social Sciences Major). Interestingly, some students felt that the *expectation* to be mindful and engaged could become an additional source of pressure: *“It’s like now we are supposed to be not just good students, but also ‘mindful’… It’s another set of expectations, and honestly, it can feel overwhelming…”* (Participant 10, Female, Sophomore, STEM Major).

In essence, the qualitative data provide a rich, nuanced understanding. Students experience flow through challenge and motivation, use EI for resilience, and employ mindfulness for focus and calm. These resources are valuable but interact with—and can be limited by—factors like intrinsic interest, external stressors, and even the pressure associated with self-improvement itself. These insights highlight the complex nature of student engagement and point to the need for holistic support strategies.

## 5 Discussion

This multi-phase mixed-methods study explored how academic flow, trait emotional intelligence (EI), and mindfulness interrelate with academic engagement among Chinese university students. Our findings confirm direct positive links between flow, EI, and engagement at the trait level, and importantly, establish mindfulness as a significant mediator in these relationships. The addition of Experience Sampling Method (ESM) data provides novel insights into the dynamic, in-situ operation of these constructs. This discussion interprets these multi-level results in light of existing literature and considers their implications.

The strong positive link found between academic flow and academic engagement at the trait level (*β* = 0.38; total *β* = 0.62) aligns with Csikszentmihalyi’s (1988) theory, which emphasizes deep task immersion for optimal learning (Csíkszentmihályi, 1996; Shernoff, 2012). This link may be due to flow’s ability to enhance intrinsic motivation and sustained focus (Csikszentmihalyi, 2014; Shernoff et al., 2003). Our quantitative data confirmed this, while initial qualitative findings revealed flow often occurred during intrinsically motivating tasks (Asakawa, 2010) and depended on an optimal balance between challenge and skill (Csikszentmihalyi, 2014; Fong et al., 2015; Harris et al., 2023; Mills and Fullagar, 2008). This trait-level relationship is mirrored in daily academic life, as our ESM findings demonstrated that students reported significantly higher momentary academic flow when engaged in solitary study and in conducive environments like the library, suggesting that focused, self-directed learning situations may be particularly conducive to achieving these immersive states. The importance of task characteristics identified qualitatively is further underscored by the ESM data showing students experienced greater flow when their momentary sense of skill matched the perceived challenge of their immediate task. This supports designing appropriately challenging learning activities (Buil et al., 2019; Wu et al., 2021). Beyond engagement, literature also connects flow to academic satisfaction and wellbeing (Asakawa, 2010; Guan, 2013; Joo et al., 2014; Park and Lee, 2018; Wang et al., 2020; Whalen, 1998).

Regarding emotional intelligence, our finding that trait EI directly predicts academic engagement (*β* = 0.29) is consistent with research showing that students with higher EI manage academic stress more effectively (Ferrando et al., 2011; Mavroveli and Sánchez-Ruiz, 2011) and exhibit greater persistence (Okwuduba et al., 2021; Qualter et al., 2012). This predictive relationship may stem from EI’s role in helping students regulate negative emotions that can disrupt engagement (MacCann et al., 2020). Our qualitative data corroborated this. The significance of trait EI is further illuminated by its daily manifestations: ESM results revealed that students with higher trait EI not only reported more effective momentary emotional regulation and lower average momentary stress but, crucially, trait EI also buffered the negative impact of momentary stress on their engagement. This suggests a dynamic, protective role where trait EI equips students to better handle daily academic emotional challenges in real-time. Furthermore, EI supports academic success by fostering positive interpersonal relationships (Estrada et al., 2021; Iqbal et al., 2022; Perera and DiGiacomo, 2015) and enhancing resilience (Maguire et al., 2017). This evidence (MacCann et al., 2020; Perera and DiGiacomo, 2013; Sanchez-Ruiz et al., 2013) highlights the importance of cultivating EI (Maguire et al., 2017).

A key contribution of this study is elucidating mindfulness’s central role, as demonstrated by its direct effect on academic engagement (*β* = 0.42) and its partial mediation of both academic flow (*β* = 0.24) and trait EI (*β* = 0.21) effects on engagement in our SEM analysis. The ESM findings provide compelling micro-level evidence for these pathways. We observed that momentary mindfulness was a strong contemporaneous predictor of both momentary flow and engagement, and importantly, higher momentary mindfulness at one time point predicted increased flow and engagement at the subsequent time point. This temporal precedence in daily experiences offers a dynamic lens through which to understand how trait mindfulness likely exerts its beneficial effects. Students with higher trait mindfulness also consistently reported higher state mindfulness, suggesting a stable tendency translating into more frequent mindful moments.

Qualitative data further supported mindfulness’s role, with students describing how it improved concentration (facilitating flow) and helped manage academic pressures (linking to EI and engagement). The mediating role of mindfulness in the flow-engagement link (Ergas and Hadar, 2019) is likely enacted through the sharpened focus and reduced distraction observed in moments of higher state mindfulness (Brown and Ryan, 2003; Roeser, 2014), as indicated by our ESM results. For the EI-engagement pathway, the ESM finding that effective momentary emotional regulation attenuated the negative impact of stress on engagement, coupled with trait EI predicting better momentary regulation, suggests that mindfulness may contribute by enhancing this capacity for adaptive emotional responding in the moment (Hill and Updegraff, 2012; Roemer et al., 2015). Indeed, the cross-level interaction where higher trait EI strengthened the stress-buffering effect on engagement further supports this dynamic interplay. While literature extensively documents mindfulness’s benefits for concentration, stress reduction, emotional resilience, and cognitive functions (Aherne et al., 2016; Baumgartner and Schneider, 2021; Bush, 2013; Cásedas et al., 2020; Chiesa et al., 2011; Eberth and Sedlmeier, 2012; Grossman et al., 2004; Kabat-Zinn, 2003; Kerrigan et al., 2017; Khoury et al., 2013; Lertladaluck et al., 2021; Maynard et al., 2017; Miralles-Armenteros et al., 2021; Morone et al., 2012; Teper et al., 2013; Vidal-Meliá et al., 2022; Vorontsova-Wenger et al., 2021; Whitfield et al., 2022; Xie, 2022; Yeh et al., 2019), our ESM data provide a novel window into *how* these benefits unfold in real-time within students’ academic lives, demonstrating its immediate positive association with adaptive academic states.

In conclusion, this multi-phase, mixed-methods study, integrating survey, ESM, and qualitative data, powerfully underscores mindfulness as a pivotal asset in academic settings (Brown and Ryan, 2003; Kabat-Zinn, 2003; Sauer et al., 2013). By cultivating present-moment awareness (Chems-Maarif et al., 2025; Creswell, 2017; Shapiro et al., 2006), students appear better equipped to handle stress, maintain focus, enter flow states, and regulate emotions effectively, not just as general traits but as dynamic, momentary capacities. The convergence of our SEM findings showing mindfulness as a key mediator, with ESM data illustrating its real-time links to flow and engagement and its role in adaptive emotional responses, provides a more comprehensive understanding of its importance. These results highlight its increasing prominence in education for supporting student wellbeing and learning outcomes (Felver et al., 2016; Schonert-Reichl and Roeser, 2013; Tomlinson et al., 2018; Zhang et al., 2024).

## 6 Conclusion

This study sought to answer the central research question: To what extent does mindfulness function as a psychological mechanism that mediates the relationship between academic flow, emotional intelligence, and academic engagement? In direct response, our multi-phase, mixed-methods findings demonstrate conclusively that mindfulness is a critical and significant mechanism.

Our results confirmed our primary hypotheses. The trait-level analysis (SEM) supported our hypothesis that mindfulness partially mediates the positive relationships that both academic flow and trait EI have with academic engagement (H1–H4). This was further substantiated by our momentary-level data (ESM), which confirmed that fluctuations in mindfulness predicted subsequent changes in flow and engagement in students’ daily lives (H5–H6). Moreover, the data supported our hypotheses regarding the protective role of EI in buffering the effects of daily stress (H7–H10).

The primary relevance of this research to the field is its provision of integrated, multi-level evidence that moves beyond correlation to illuminate a key psychological process. By combining trait-level modeling, momentary assessments, and qualitative insights, this study establishes mindfulness not just as a beneficial correlate but as an actionable, dynamic skill that underpins student engagement. These findings provide a strong empirical foundation for developing targeted interventions to foster the psychological resources necessary for academic success and wellbeing.

## 7 Implications, limitations, and future directions

Our multi-phase mixed-methods findings offer practical implications for enhancing student engagement, particularly within the demanding, collectivist context of Chinese higher education and similar settings. The convergence of trait-level relationships (SEM) and dynamic, in-situ experiences (ESM) suggests that targeted interventions focusing on academic flow, EI, and mindfulness can foster deeper student engagement.

First, given mindfulness’s significant mediating role in the SEM and its observed benefits on momentary flow and engagement in the ESM phase, integrating mindfulness practices appears especially beneficial. Such interventions could enhance students’ ability to concentrate and manage emotional responses to intense academic pressures. The ESM data, showing momentary mindfulness predicting subsequent adaptive states, support the utility of brief, accessible practices that students can deploy *in-situ* (e.g., short meditations before study, mindful attention during lectures), potentially guided by digital prompts or integrated into learning platforms. Practical approaches include offering Mindfulness-Based Stress Reduction (MBSR) programs, incorporating these brief exercises into classes, adding such strategies to study workshops, or using popular digital platforms for delivery.

Second, the findings support incorporating EI training into student development. The ESM results, demonstrating how higher trait EI buffers the negative impact of momentary stress on engagement and correlates with more effective momentary emotional regulation, provide strong evidence for interventions that build practical skills applicable to immediate academic challenges. Within the Chinese context, these programs could focus on enhancing emotional clarity, regulation, and interpersonal skills, helping students navigate peer competition and build supportive relationships. Equipping students with EI skills can foster resilience and improve wellbeing amidst significant academic and social pressures.

Finally, educators should design learning environments that promote academic flow. Our qualitative data confirm that Chinese students experience flow when tasks are optimally challenging and personally meaningful. The ESM findings, highlighting higher momentary flow during solitary study and in specific locations like libraries, can inform more precise recommendations to students about optimizing their study environments and routines. Therefore, educators can boost engagement by creating activities (e.g., project-based learning with clear goals, autonomy, and timely feedback) that align with students’ intrinsic motivations and career interests. This approach may also help prevent academic burnout.

Despite its multi-level insights, this study has limitations that open avenues for future research. First, while the ESM component provided longitudinal micro-level data over a week, the initial quantitative phase establishing trait-level relationships was cross-sectional, precluding definitive causal inferences at that level. Future research should employ broader longitudinal designs to track trait-level changes and the long-term impact of interventions over entire semesters or academic years. Second, our sample was limited to Chinese university students, which restricts the generalizability of our findings. Cultural and institutional factors can influence these psychological relationships. Therefore, replications in diverse cultural contexts—both within China (e.g., comparing different university types or regions) and internationally—are essential to distinguish universal patterns from culturally specific ones.

Third, our reliance on self-report measures for both the initial surveys and the ESM phase, though validated, carries a risk of social desirability bias, potentially heightened by cultural norms. While ESM captures in-the-moment experiences, reducing recall bias, the ratings are still subjective. Future studies would benefit from integrating objective data, such as academic performance records, Learning Management System (LMS) analytics, or even physiological markers (e.g., heart rate variability during ESM assessments of stress) to triangulate findings more robustly. Fourth, the ESM protocol itself, while providing rich data, has inherent limitations such as participant burden and potential reactivity to repeated measurements. Future ESM studies could explore varied prompting schedules (e.g., event-contingent triggers for reporting flow or stress), incorporate passive sensing data from smartphones to complement active self-reports, or utilize a broader array of validated ultra-brief momentary measures.

Finally, while we confirmed mindfulness as a key mediator in our trait-level model, and explored momentary dynamics via ESM, other variables likely play a role. Future research should explore additional mediators and moderators pertinent to the Chinese context, such as personality traits, culturally specific values, teaching styles, peer dynamics, and institutional support. Moreover, advanced analytical techniques, such as dynamic structural equation modeling integrating trait and ESM data, or person-centered analyses (e.g., Latent Profile Analysis based on ESM patterns), could offer even deeper insights into different typologies of student experiences and the complex interplay of these factors over time.

## Data Availability

The data analyzed in this study is subject to the following licenses/restrictions: the datasets generated and analyzed during the current study are available from the corresponding author on reasonable request. Requests to access these datasets should be directed to Rui Ma, mauri19840213@sina.com.
